# Using Data-Driven Model-Brain Mappings to Constrain Formal Models of Cognition

**DOI:** 10.1371/journal.pone.0119673

**Published:** 2015-03-06

**Authors:** Jelmer P. Borst, Menno Nijboer, Niels A. Taatgen, Hedderik van Rijn, John R. Anderson

**Affiliations:** 1 Carnegie Mellon University, Dept. of Psychology, Pittsburgh, United States of America; 2 University of Groningen, Dept. of Artificial Intelligence, Groningen, the Netherlands; 3 University of Groningen, Dept. of Psychology, Groningen, the Netherlands; Max Planck Institute for Human Cognitive and Brain Sciences, GERMANY

## Abstract

In this paper we propose a method to create data-driven mappings from components of cognitive models to brain regions. Cognitive models are notoriously hard to evaluate, especially based on behavioral measures alone. Neuroimaging data can provide additional constraints, but this requires a mapping from model components to brain regions. Although such mappings can be based on the experience of the modeler or on a reading of the literature, a formal method is preferred to prevent researcher-based biases. In this paper we used model-based fMRI analysis to create a data-driven model-brain mapping for five modules of the ACT-R cognitive architecture. We then validated this mapping by applying it to two new datasets with associated models. The new mapping was at least as powerful as an existing mapping that was based on the literature, and indicated where the models were supported by the data and where they have to be improved. We conclude that data-driven model-brain mappings can provide strong constraints on cognitive models, and that model-based fMRI is a suitable way to create such mappings.

## Introduction

Formal models constitute one of the dominant methodologies in cognitive science: they played a major role in more than half the articles published in the Cognitive Science journal in 2013 (53% of the articles in Cognitive Science volume 37 mentioned ‘model’, ‘simulation’, or ‘computational theory’ in their abstract.). However, the approach is not without its problems, as the quality of models is notoriously hard to evaluate [1–3]. Although there have been several proposals on how to test cognitive models [4–6], no consensus has been reached. This is partly due to the wide variety of models; it is for instance unclear if and how approaches suitable for mathematical models could be extended to symbolic process models.

**Fig 1:**
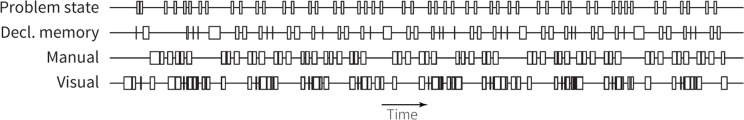
Cognitive operations in one trial of a model of multitasking. Such a trial involved 20 responses [8]. The four rows indicate four model components; boxes indicate when a component was active.

One important modeling requirement—shared by the different proposals—is that a model should be able to predict data of new experiments, for instance reaction times and accuracy (also referred to as *generalizability* or *applied approach* [2,5–7]). However, even if models are capable of predicting behavioral data, their complexity often exceeds constraints provided by behavioral data. For example, Fig. 1 shows cognitive operations assumed by a model of multitasking behavior, for one trial of the task (which involved 20 responses [8]). In that model the critical activities where updating a working memory problem state, retrieving information from declarative memory, performing various visual encodings, and outputting the response. Any model that outputs the same responses at the same time would make equivalent behavioral predictions. While not necessarily easy, one could imagine re-arranging the components (note that we use ‘components’ to refer to concepts of a computational model in this paper) or inserting completely different intervening processes in ways that would leave the output unchanged.

To provide additional constraints for cognitive models, researchers have turned to neuroscience (e.g., [9,10–12]). A prime example of this is the ACT-R cognitive architecture (Adaptive Control of Thought-Rational; [13]). ACT-R is a general psychological theory, but it also provides a simulation environment in which task models can be developed. It thereby ensures that theoretical ideas have to be formally specified, giving them additional credibility [14]. It has been used extensively both in basic psychological research as well as in more applied settings (e.g., cognitive tutors [15]; see act-r.psy.cmu.edu for over 1000 papers that use or discuss ACT-R). After a development based on behavioral and eye-tracking data that extends back to the 1970s, in 2003 a mapping was developed from components of the architecture to brain regions [16–18]. Since then, models developed in ACT-R automatically predict the fMRI BOLD response in several regions of the brain, and can thus be tested and constrained by fMRI data [9,19,20]. This approach has been extremely fruitful (act-r.psy.cmu.edu lists over 60 papers that use or discuss this approach), and was one of the main driving forces behind the latest version of the architecture [13].

Before neuroimaging data can be used to constrain a computational model, one needs a mapping from model components to brain regions. In the case of ACT-R, the initial mapping was based on a reading of the literature [16,21], and adapted slightly based on experience with new tasks. This approach is suboptimal—in the sense that it is subjective—but it was the best option at the time. In this paper we will propose and demonstrate a new, formal method to create such a mapping: model-based fMRI analysis. Model-based fMRI analysis shows the most likely location of model components in the brain by calculating the correlations between activity of certain model-components (or settings of certain model parameters) and observed brain activity [11,22,23], and can thus form the basis for a mapping of model components to brain regions. As model-based fMRI is a formal, data-driven method, it is to be preferred over the original, subjective approach for creating model-brain mappings, to prevent researcher-based biases. The goal of the current paper is to show that it performs at least as well as the original method, and that it is therefore a suitable substitution for the original, subjective method.

Because the results of model-based fMRI are dependent on the quality of the model that is used in the analysis, we will use a recent model-based meta-analysis of five tasks with associated ACT-R models as the basis of our data-driven mapping [24]. We will subsequently apply both the original literature-based mapping and the new, data-driven mapping to two independent datasets. For each dataset we will use a model from previous research, and either optimize this model to fit the behavioral data of the current task (dataset 1) or use *a priori* predictions based on previously identified model parameters (dataset 2). Next, we will use both models to generate predictions about the patterns we expect to observe in the neural data. We will then compare these predictions to the neural data of the two model-brain mappings. Summarizing, we use two datasets, for each dataset we use a single model to generate neural predictions, and we compare these predictions to data of two different model-brain mappings, the original mapping and the new, data-driven mapping.

The first dataset consists of a relatively simple algebra task, and should—given our experience of modeling such tasks—result in a good fit of the data. For this dataset we merged two existing models into a new model. The second dataset is a much more challenging multitask for which we made *a priori* predictions using a previously published model. We do not expect to match all details of the data in this case, but we will use it as an example of how the method can direct the development of cognitive models.

In the remainder of the introduction we will first discuss how ACT-R models can be used to predict fMRI data, and how such data can be used to constrain models. Next, we will describe model-based fMRI analysis, as well as the meta-analysis on which the new mapping will be based.

### Predicting and evaluating ACT-R models with fMRI

**Fig 2:**
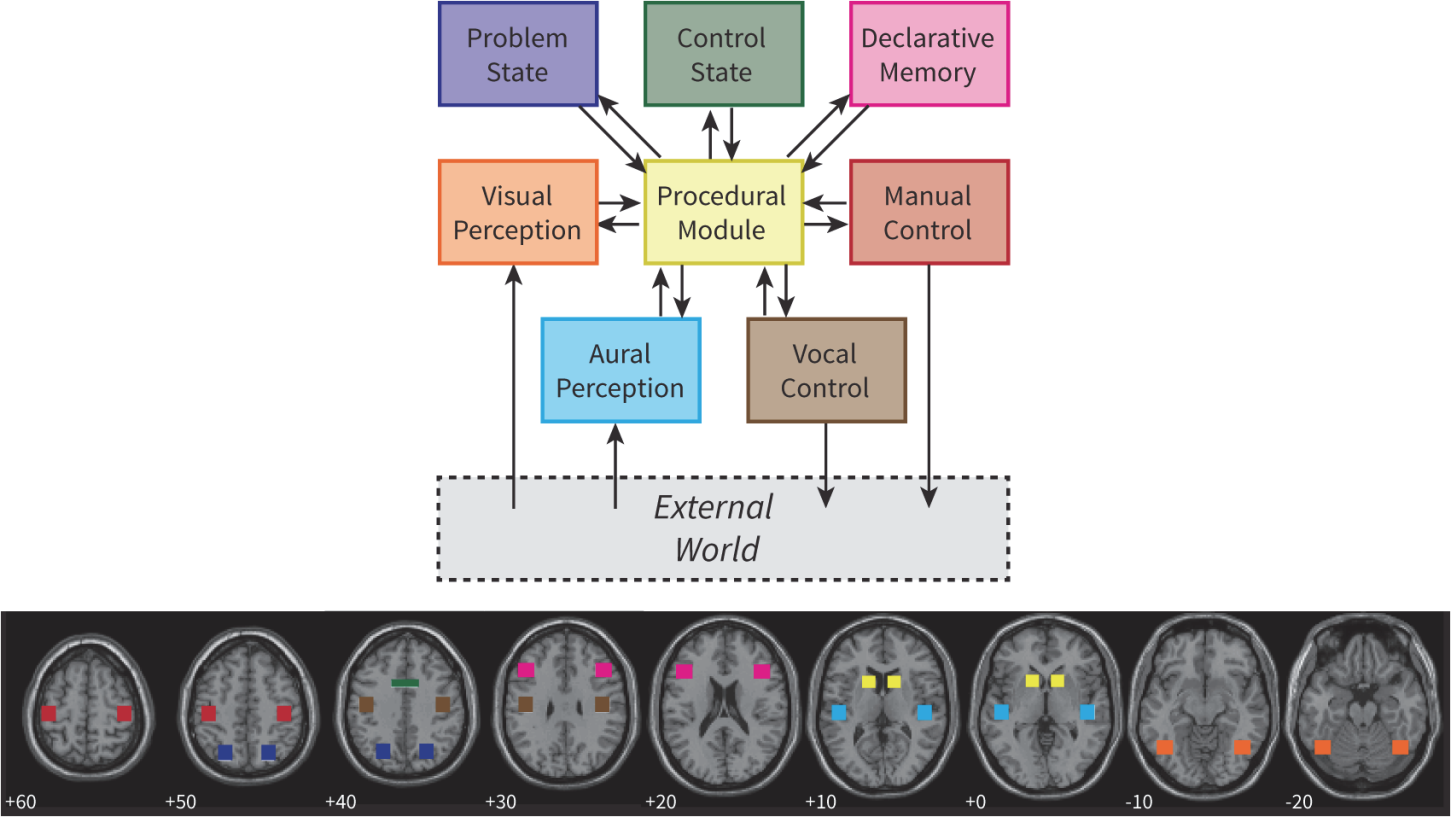
The cognitive architecture ACT-R and its mapping on brain regions. Numbers indicate the z-coordinate of each slice (MNI coordinates). For illustrative purposes only, similar to Fig. 1 in [20].

Fig. 2 gives an overview of the ACT-R architecture and its current mapping to brain regions ([13,20]; see Table 1 for details). ACT-R assumes that cognition emerges from the interplay of several independent components, which are typically referred to as modules. Two modules take care of input, the visual and aural modules, and two modules perform actions, the manual and vocal modules. To perform cognitive operations, ACT-R has four additional modules: declarative memory, procedural memory, the problem state, and the control state. The modules interact through buffers of limited size with the procedural module. The procedural module contains rules that can fire based on the contents of the buffers. For instance, if the visual module has encoded an equation from the screen, a rule can fire that initiates the retrieval of an arithmetic fact from declarative memory. To create an ACT-R *model* one has to write such rules and specify the model’s knowledge (e.g., ‘3 + 4 = 7’) in declarative memory. ACT-R itself can therefore be seen as a theory of the fixed architecture of the mind, while a model is dependent on the task that is simulated, and can be seen as a sub-theory specifying how a particular task is performed.

**Table 1:** Regions-of-interest in the original mapping and in the new data-driven mapping.

	Original Mapping		Data-driven mapping	
Module	Brain Region	Coordinates	Name	Coordinates
Problem State	Intraparietal sulcus	−24, −67, 44	Intraparietal sulcus	−38, −50, 48
Declarative Memory	Inferior frontal sulcus	−43, 24, 25	Inferior frontal sulcus	−46, 16, 26
Manual	Precentral gyrus	−42, −23, 54	Precentral gyrus	−33, −18, 57
Aural	Superior temporal gyrus	−48, −21, 7	Superior temporal gyrus	−58, −21, 4
Visual	Fusiform gyrus	−43, −60, −16	Middle occipital gyrus	−30, −84, 15

*Note*: Coordinates are for the left hemisphere (MNI).

As mentioned above, ACT-R models predict the blood-oxygen-level dependent (BOLD) response that is measured with fMRI [9]. Each module is associated with a small region of the brain, shown in Fig. 2. The assumption is that this region is active when the corresponding module is active. For instance, when the manual module issues a key-press, the associated area in the motor cortex is assumed to show an increased BOLD response. Note that it is not claimed that these are the only regions that are active in response to the modules. The claim is that these regions are necessarily active when the associated modules are active, and thus provide good indicators of module activity. More formally, we describe the activity of a module as a 0–1 demand function. This function is convolved with a hemodynamic response function (HRF), which describes the BOLD response (e.g., [25–27]). In the current paper we used the HRF of the SPM analysis software [28], which is the difference of two gamma functions.

**Fig 3:**
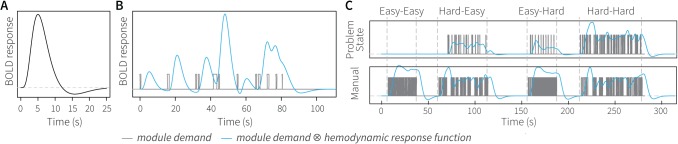
Convolving module activity with a hemodynamic response function. (a) HRF, (b) convolving short periods of module activity with the HRF, and (c) example of the problem state module and manual module for four conditions of a multitasking experiment [8]. For illustrative purposes only, similar to Fig. 3 in [72].

Fig. 3 illustrates this process (for more details, including example model code in Lisp and convolution code in Matlab, see [20]). Panel A shows the HRF in response to neural activity at time 0. The HRF increases slowly, and only reaches its peak around 5 seconds after the neural activity. Fig. 3B shows the result of convolving a demand function (gray) with the HRF. The predicted BOLD response depends on the amount and duration of periods of module activity. In essence, for each period of activity an HRF is assumed, which are summed to predict the final response [9]. Fig. 3C shows the results of this process when applied to two modules of a complex model (cf. Fig. 1; [8]). The figure depicts four conditions from left to right, which increase in difficulty. Whereas the manual module predicts a lower BOLD response for the more difficult conditions (because the same amount of key-presses are spaced out over more time), the problem state module predicts a strong increase in activity with task difficulty. These predictions can be compared to fMRI data, and can thus be used to evaluate ACT-R models (we will discuss two examples in this paper). We will refer to this type of analysis as a region-of-interest (ROI) analysis.

If one has a mapping from model component to brain regions, such an ROI-analysis brings strong constraints to a model: not only does the model have to fit behavioral data, but it also has to match the BOLD response in several brain regions over time. However, the analysis depends on having a correct mapping between model and brain regions, a mapping that is often based on a reading of the literature, and thus inherently subjective. In this paper we propose to use a more objective model-based fMRI analysis to create such a model-brain mapping.

### Model-based fMRI

Model-based fMRI is a recent analysis technique that uses computational models to analyze fMRI data (e.g., [20,22,23]). The goal of model-based fMRI is to find the most probable location of model components in the brain. The basic idea is to regress predictions derived from a computational model against brain data, thereby showing which brain regions correlate significantly with the model predictions, and are thus most likely to implement the functionality of the model component. This analysis is very similar to standard fMRI analysis, in which the condition-structure of the experiment is regressed against brain data, indicating which regions correspond significantly to the conditions of the experiment (e.g., Friston et al., 2007). Thus, model-based fMRI analysis can be used to create model-brain mappings in a data-driven manner.

Model-based fMRI can be used in combination with a variety of models, which are typically developed based on behavioral data. In most cases, parameter values of mathematical models have been used as the regressors representing the model. Such models have for instance been used to locate regions involved in reinforcement learning [29–32], category learning [33], and decision making [34,35]. In 2011, we have shown that it is also possible to use predictions derived from a symbolic process model as input for model-based fMRI [36]. Analogous to the ROI-analysis, we took the module activity of an ACT-R model and convolved it with a hemodynamic response function (Fig. 3). The resulting predictions were regressed against fMRI data, indicating the locations of the ACT-R modules. Recently, a similar method has been used to map the activity of ACT-R modules on EEG oscillatory power [37].

Although model-based fMRI is in principle an extremely powerful method to map the functionality of the brain [11], the results critically depend on the quality and assumptions of the model. To avoid idiosyncrasies of particular tasks and models, Borst and Anderson [24] applied model-based fMRI to five previously published datasets with associated ACT-R models, and subjected the results to a meta-analysis. As their tasks ranged from paired associates [38,39] and algebra [40] to information processing and multitasking [8,41], we can be reasonably sure that the results are task-independent. Borst and Anderson determined the location of five ACT-R modules: the aural, visual, and manual modules, as well as declarative memory and the problem state module. We will now briefly review their results, as we will use these results as the basis of our new data-driven model-brain mapping.

As expected, the aural module was localized in the superior temporal gyrus, an area involved in speech processing [42]. The visual module correlated most strongly with activity in the left and right occipital gyri, which are involved in spatial-visual attention (e.g., [43]). ACT-R’s right manual module—all responses were made with the right hand in the tasks that were analyzed—mapped onto the left motor cortex, extending from the premotor cortex into the parietal lobe. Both declarative memory and the problem state module (ACT-R’s working memory, which is comparable to the focus of attention in recent theories, e.g., [44–47]) correlated significantly with activity around the inferior frontal gyrus and in the anterior cingulate. In addition, the problem state module also correlated with a large region around the intraparietal sulcus (together, these regions form the fronto-parietal network; [48–52]). To dissociate those two modules further, it was investigated in which regions the declarative memory predictors explained more variance than the problem state predictors, and vice versa [24]. This resulted in a region in the inferior frontal gyrus for declarative memory, and an exclusive problem state region around the intraparietal sulcus. In the next section we will use these results as the basis for our new model-brain mapping.

## Method: Creating a New Model-Brain Mapping

The regions that have been associated with the modules of the ACT-R architecture vary in size from 60 to 100 voxels. Using the results from Borst and Anderson [24] we created a set of comparably sized regions. In this section we will explain how we combined those results with anatomical constraints and discriminability considerations to define a set of similarly sized regions. In the next two sections of the paper we will apply the new mapping, as well as the original literature-based model-brain mapping, to two new datasets. We will discuss the methods related to those datasets in their respective sections.

To create the new mapping, the results of the model-based fMRI meta-analysis were thresholded with *p* < 10^–7^ and at least 250 voxels per cluster (following [24]). For the visual and aural modules, we then took the most significant voxel as a seed, and iteratively selected the most significant voxel bordering the currently selected region, until we reached regions consisting of 100 voxels. For the manual module, we did not use all results from the model-based meta-analysis since some active regions were outside of primary motor areas. Instead, we restricted the definition to voxels in the precentral gyrus (as defined in the AAL atlas [53]). It is well known that the primary motor cortex is located in this region, which matches the functionality of the manual module. Because only 80 significant voxels remained, this ROI is slightly smaller than the others.

For the problem state and declarative memory modules we followed the same procedure, except that we took the most significant voxel of the dissociation analysis as a seed as both modules resulted in partially overlapping activation patterns. That is, for the problem state module we used a voxel close to the intraparietal sulcus and for declarative memory a voxel close to the inferior frontal gyrus. These voxels dissociated optimally between problem state and declarative memory contributions to the BOLD signal, and will consequently lead to regions that can be used most successfully to distinguish and constrain these modules.

**Fig 4:**
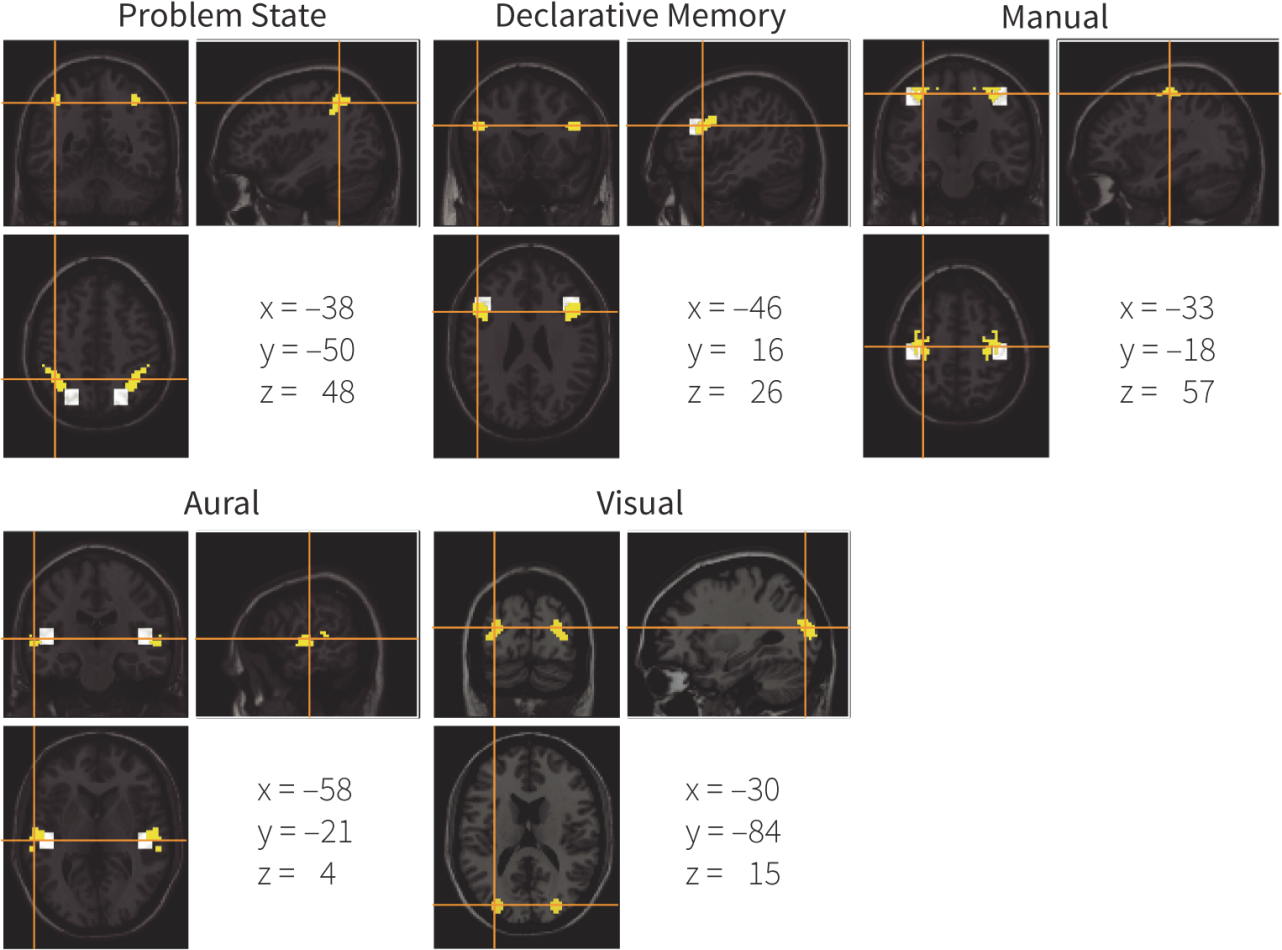
The new mapping of ACT-R modules to brain regions. Yellow indicates the new mapping, white the original one (the original visual ROI is not shown because it is located on different slices—its center is −43, −60, −16). Coordinates are the center of mass in the MNI system.

The regions that we created in this manner were all left-lateralized because the most significant voxels were located in the left hemisphere. To create right-hemisphere homologues, we mirrored these regions. The resulting ROIs are shown in Fig. 4, in yellow, and summarized in Table 1. The white squares indicate the original mapping of ACT-R (the original visual ROI is not shown, as it is located outside the displayed slices). For most modules the original and the new mapping overlap in part. However, the new region for the problem state is more anterior than in the original mapping, and the new visual region is in a completely different location than the original ROI (located in the fusiform gyrus; *x* = −43, *y* = −60, *z* = −16). In addition, unlike the original mapping, the new mapping follows brain structures, which might increase the power of the analysis (assuming that brain functions do not cross structural boundaries). The new ROIs can be downloaded from http://www.jelmerborst.nl/models, both as binary images and as MarsBar ROI definitions for use with the SPM analysis software [54].

To investigate the power of the new mapping, and thereby the suitability of model-based fMRI as a basis for such a mapping, we will apply both the new and the original model-brain mapping to two datasets. For dataset 1 we will develop a model, and fit this model to the behavioral results of the dataset. For dataset 2 we will take a previously published model, and use it to generate *a priori* predictions. Next, we will generate neural predictions from both models, and compare these predictions to the neural data of both the new, data-driven model-brain mapping and the original mapping.

## Dataset 1: Algebra

The first dataset that we considered concerns relatively simple algebra problems. This dataset was previously used to investigate metacognitive activity in mathematical problem solving, by comparing regular problems to so-called exception problems [55]. To investigate the new ROIs we will only look at the regular problems, as we have a relatively good understanding of how these are solved. To account for the subjects’ behavior, we combined two previously developed models of this task [13,56]. Given our experience with algebra tasks in general and this task in particular [40,57,58], we expected our model to give a good account of the data, and therefore to provide a good test of whether the new ROIs reflect the activity of the model’s resources.

### Design

In the experiment subjects were asked to solve ‘pyramid’ problems. A pyramid problem takes the form of *base* $ *height* = *value*, for instance 4 $ 3 = 9. To solve a pyramid, one has to perform repeated addition: 4 $ 3 = 4 + 3 + 2 = 9, where the *base* indicates the starting value and the *height* the number of terms to add (where each term is one less than the previous term). In the experiment, either the *base*, *height*, or *value* was unknown. Consequently, subjects were asked to solve these three kinds of pyramid problems:

- Solve-for-value: 4 $ 3 = *x*
- Solve-for-height: 5 $ *x* = 9
- Solve-for-base: *x* $ 4 = 26

The *base* of the problems ranged from 4 to 9 and the *height* from 2 to 5, resulting in *values* from 4 $ 2 = 7 to 9 $ 5 = 35. We will analyze the problems based on whether they had a small (4–6) or a large (7–9) base and whether they had a small (2–3) or a large (4–5) height. Each subject solved 72 problems in the fMRI scanner, 18 problems per condition. Subjects had 30 seconds per problem to calculate a solution; to enter a response they used a mouse to click on an on-screen numeric keypad, finishing a response by clicking a submit button. After responding subjects immediately advanced to a 5s feedback screen, followed by 12 seconds of repetition detection. Then the next trial started with a fixation screen for 4 seconds, followed by the next pyramid problem. In total, twenty subjects participated in the experiment. Detailed methods are reported in [55].

### Model

To account for results of similar experiments, two slightly different models were developed on the basis of behavioral data [13,56]. We combined these models into a single model, to which we will refer as the behavioral model, to simulate the behavior in the current experiment. The model is available for download on http://www.jelmerborst.nl/models, under the title of this paper. The model starts by encoding a problem from the screen: it attends the *base*, *height*, and *value* in turn, and while doing so follows its eye gaze with the mouse cursor [59,60]. Based on which variable is unknown, it proceeds down one of three solution paths. If the *value* is unknown, the model starts an iterative addition sequence, starting with adding the *base* to the *base − 1*, and finishing when it has made as many additions as the *height* indicated. For each addition it retrieves the solution from declarative memory, which takes a certain amount of time (on average 483 ms, estimated from the behavioral data). It then continues to the response phase. If the *height* is unknown, it starts the same iterative addition process, but terminates this process when the result matches the *value*, and reports the number of additions. Finally, if the *base* is unknown, the model follows a guess-and-check procedure (as did the subjects; [55]). It first guesses a number to fill in as the *base*, and then follows the *base*-procedure to check if this guess leads to the right *value*. If it does the model reports the guessed *base*. If the resulting value is too small, the model reports the guessed *base* + 1, if it is too big it reports the guessed *base* − 1. After determining the response the model looks at the screen to find the right button. It then moves the mouse cursor to this button, clicks the button, reads the entered response, and continues to the next button. After entering the full response in this manner, the model clicks the submit button, and reads the feedback to determine whether it was correct.

The total time the behavioral model needs to complete a trial is the sum of the encoding steps, the addition steps, and the response steps. The times for encoding and responding are fairly constant for the different trial types, but the number of addition steps—and thus the total time spent on addition—varied considerably (range 1.20–29.65 s, m = 5.62 s, sd = 2.86 s). First, larger heights lead to more addition steps and thus to a longer RT. Second, larger bases result in more carries than smaller bases. For instance, compare 4 $ 3 = 4 + 3 + 2 = 9 to 9 $ 3 = 9 + 8 + 7 = 24. In the second case two carries have to be performed, which add additional computations to the model and therefore also result in longer RTs. To fit the response times of the model to the behavioral data, we manually adjusted the activation level of the addition facts (e.g., ‘3 + 4 = 7’) in declarative memory (higher activation levels lead to faster retrievals and vice versa, [13]).

### Analysis

To investigate the new ROIs we used correct trials only. Two subjects solved respectively 49% and 76% of the regular trials correctly, where all other subjects scored >85%. We excluded these subjects from the analysis, leaving 18 subjects that solved 93.4% (SE = 0.9%) of the problems correctly on average. In addition, we removed problems with a response time (RT) exceeding 2 standard deviations from the mean per condition and subject (4.2% of the trials).

### Behavioral results

**Fig 5:**
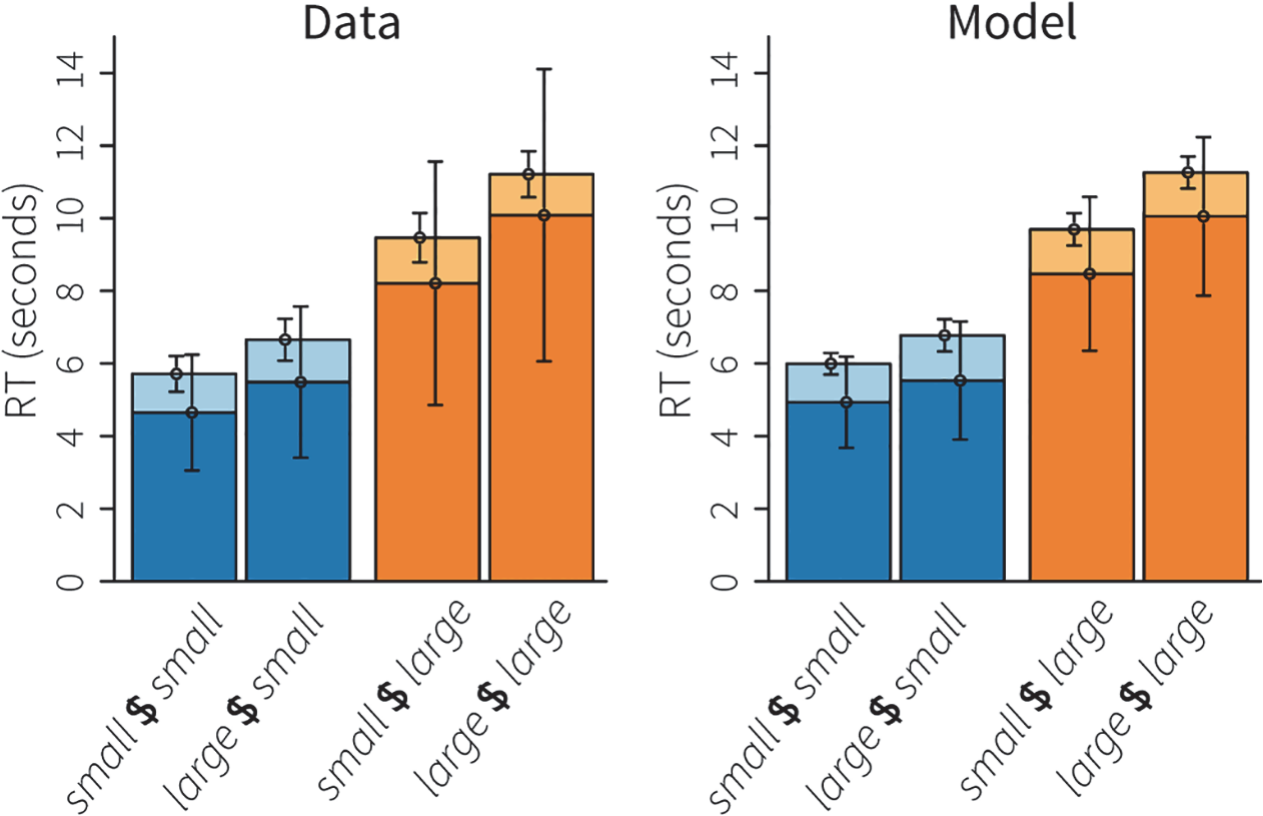
Response times of the algebra dataset. Blue bars indicate small heights, orange bars large heights; dark parts of the bars indicate the time until the first mouse click, light parts the time between the first click and clicking the submit button. Error bars indicate the average standard deviation.

Fig. 5 shows the response times, on the left for the data, on the right for the model. Height had a substantial effect on response times, with large heights (orange bars) leading to longer response times than small heights (blue bars). Given that the height determines the number of terms in the addition, this is not surprising. A repeated-measure ANOVA confirmed this effect: *F*(1,17) = 190.9, *p* < .001. In addition to height, base also had a positive effect on RTs, with large bases leading to longer RTs (*F*(1,17) = 20.5, *p* < .001). This effect is explained by the slightly larger numbers that have to be added, more often resulting in carries. Comparing the ‘cognitive phase’ (dark parts of the bars, before the first number was entered) to the response phase (light parts, first number until submit button) indicates that the effects on RT were almost completely due to the cognitive phase. In addition, the average standard deviations indicate that most of the variability was also contained in the cognitive phase. The model captured all these effects; the biggest discrepancy between model and empirical data is the lower variability in the model’s cognitive phase, especially for the large heights.

### Imaging results

**Fig 6:**
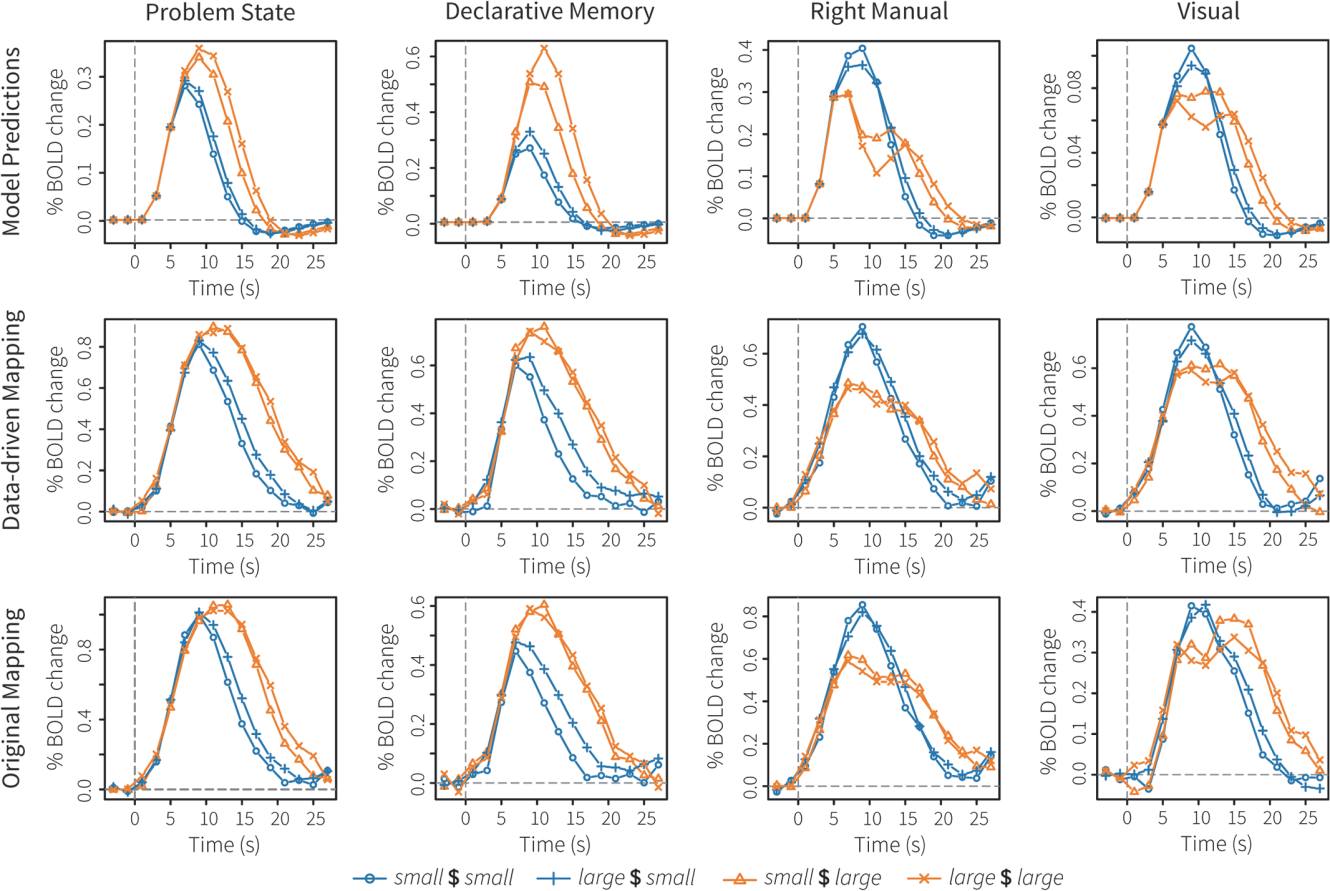
Imaging results of the algebra dataset. The top row shows model predictions, the middle row the BOLD responses in the new, data-driven ROIs, and the bottom row the BOLD responses in the original ROIs.

The top row of Fig. 6 shows the model’s BOLD predictions for four modules: the problem state module (ACT-R’s working memory), declarative memory, the right-manual module, and the visual module. The next two rows show the results in the new, data-driven ROIs and in the original ROIs, respectively. All results are from the ROIs in the left hemisphere, as these typically show the strongest effects. Table 2 reports three fit measures of the model predictions to the data: the R^2^, the root-mean-square deviation, and Tucker’s Congruence Coefficient (TCC; [61]). Although we assume the first two measures to be familiar, TCC might not be. TCC measures the proportionality of the elements in two vectors, that is, it is another way of measuring the similarity of two vectors. The values of TCC range between −1 and 1, with −1 indicating a complete opposite (with a correlation of −1), and 1 indicating identical vectors. In practice, values between .85 and .94 correspond to a fair similarity, and values over .95 indicate that the two vectors are almost identical [62]. Unlike the R^2^ measure of correspondence, TCC does take into account the slope of the vectors (positive vs. negative), the sign of the vectors, and it can handle horizontal lines. See S1 Fig. for several demonstrations. In addition to the TCC of the aggregate data, we also report the average TCC per participant, and its standard deviation and range.

**Table 2:** Fit measures for the new data-driven mapping and the original mapping on the algebra dataset.

	Data-driven mapping				Original Mapping			
Module	TCC	Ind. TCC	R^2^	RMSD	TCC	Ind. TCC	R^2^	RMSD
Problem State	.86	.79 (.10; .59–.94)	.67	.35	.87	.74 (.39; −.81–.92)	.70	.43
Declarative Memory	.91	.70 (.25; −.11–.91)	.82	.20	.91	.71 (.18; .25 –.90)	.84	.13
Right Manual	.94	.71 (.40; −.42–.93)	.89	.17	.93	.74 (.32; −.32–.91)	.87	.25
Visual	.96	.88 (.09; .57 –.94)	.93	.33	.90	.59 (.44; −.60–.86)	.69	.17

*Note*: TCC = Tucker’s Congruence Coefficient, Ind. TCC = mean individual TCC (standard deviation, range), RMSD = root-mean-square deviation.

In general, both model-brain mappings showed very similar BOLD responses, which, in turn, were similar to the model predictions. The problem state module showed the least good fit, with R^2^-values around .7 and TCC values of .86 and .87. Although the model predicted the order and the difference between conditions correctly, the activity persisted for a longer period of time than assumed by the model. This probably indicates that subjects also used their working memory to interpret the feedback, which was essentially ignored by the model as soon as it had determined that it was correct. With respect to declarative memory, the model predictions might seem less good than for the problem state module, but the fit measures indicate otherwise. The main difference was that the model predicted the *small* $ *large* problems to have a significantly lower BOLD response than the *large* $ *large* problems, while the two problem types showed an almost identical response in the fMRI data.

For the right manual module (associated with the left motor cortex), the model predicted a decrease between initial movements and entering the response for the large-height problems. Although there seems to be a similar effect in the data, it is much less pronounced than in the model, indicating that subjects kept moving the mouse around while solving the problems. However, the model correctly predicted the sometimes very small differences between conditions. Finally, for the visual module, the new data-driven ROI performed slightly better than the original ROI, in the sense that the BOLD response in the data-driven ROI was closer to the model prediction. In addition, the signal in the data-driven ROI seems a little smoother (especially scans 0–4), which might indicate that the new ROI is less susceptible to noise.

### Discussion

As expected the model accounted well for the behavioral data, and also matched the fMRI data relatively closely. There were only small differences between the original and the new, data-driven ROIs, indicating that model-based fMRI is a suitable method to create a mapping between model components and brain regions. The regions associated with the visual module showed the greatest difference, which was not surprising given that these were also the furthest apart in the brain. The new visual ROI matched the model predictions a little better than the original ROI, perhaps indicating that the new ROI should be preferred. However, this conclusion is dependent on the model being correct.

We used a relatively simple task as a first test of the data-driven model-brain mapping, in order to be reasonably sure that our model would give a good account of the data, which gave us some sort of ‘ground truth’ to compare the data to. However, the comparison normally goes the other way: it is assumed that the ROIs are good indicators of the modules of ACT-R, and they are used to test the model. We will highlight this approach with our second dataset, which was much more challenging and for which we made *a priori* predictions. In addition, the complexity of our second dataset turned out to lead to more differentiating results between the two mappings.

## Dataset 2: Multitasking

The second dataset concerned multitasking. This dataset was previously used to investigate the relationship between single-task and dual-task activation in the brain [63]. To that end, three different single tasks were used, which were additionally combined to form three different dual-tasks. Based on a behavioral pilot study, a model was developed that matched accuracy and RT data [64]. This model was used to generate *a priori* fMRI predictions for five ACT-R modules: the aural module, the visual module, the left and right manual modules, the problem state module, and the declarative memory module. Here, we will test how well this model can account for the behavioral and BOLD data of the fMRI study. This will show how these kinds of analyses can be used to indicate problems with a model, and will test how the new data-driven model-brain mapping and the original mapping compare in a more complex task.

### Design

In the multitasking experiment subjects were asked to perform three tasks: a visual tracking task, a tone-counting task, and an n-back task. The tasks were performed as single tasks and as dual-tasks, resulting in six different conditions (i.e., A, B, C, AB, AC, and BC). The tasks were identical in the single-task and dual-task conditions, except that two tasks were presented concurrently in the dual-task conditions. In the single-task conditions, all tasks were presented in the center of the screen, while in the dual-task conditions one task was presented on the left half of the screen and one task on the right.

In the visual tracking task a target dot moved randomly to the left and to the right. The subjects’ goal was to keep a circular cursor centered on this dot by pressing a ‘left’ and a ‘right’ key with their right hands. Two vertical lines on each side of the dot indicated the maximum allowed distance between target and cursor. During dual-task conditions, the tracking task was displayed on the right side of the screen.

In the n-back task a stream of 12 letters was presented on the screen. Each letter was presented for 1000 ms, followed by a 1500 ms blank. For each letter subjects had to indicate whether it was the same or different as the letter two back. They used their left hands for this. During dual-task conditions, the n-back task was presented on the left side of the screen.

Finally, in the tone-counting task, 20 tones were presented over a 30 second period. Tones could be high or low, subjects were instructed to count high tones only (10–17 per trial). During this task a fixation cross was presented on the screen; subjects were asked to enter their response at the end of the trial by incrementing a counter. The tone-counting task was presented on the right side of the screen when it was performed in combination with the n-back task; it was presented on the left side of the screen in combination with the tracking task. Subjects used their right hands to respond to the counting task in the single-task condition and in combination with the n-back task; they responded with their left hands in combination with the tracking task.

All trials lasted 30 seconds, with an additional 10 seconds in the tone-counting conditions to enter a response. 19 subjects participated in the experiment; each subject completed 72 trials, 12 per condition. Detailed methods can be found in [63].

### Model

To account for the behavior on these tasks a model was developed and fit to the data of a behavioral experiment with a similar design [64]. The model was developed in ACT-R, and incorporates the ideas of threaded cognition theory [65,66] to handle multitasking situations.

To perform the visual tracking task, the model uses the visual module to perceive the locations of the circular cursor and the target. If the target has moved away from the cursor, the model presses the required key to move the cursor towards the target. It repeats this procedure until the cursor is on top of the target. To count the tones in the counting task, the model uses the aural module to listen to the tones. If a tone is high, it updates a counter in the problem state module, which thus keeps track of the total number of high tones. At the end of a trial this counter is used to enter the total number of high tones. Finally, to perform the n-back task, the model uses the visual module to encode the stimuli. It stores the last letter in the problem state module and the two-back letter in declarative memory based on the observation that the problem state module can only contain a single chunk of information [44]. When it perceives a new letter, it retrieves the letter two-back from memory and compares them. Based on whether the letters are the same or different, the model gives the required response.

Performing the single tasks in this way is relatively easy. However, the dual-task situations are more demanding. The modules of the ACT-R architecture operate in parallel, but each module itself proceeds serially [67]. This results in interference between tasks when they require the same resources at the same time (e.g., [66]). For example, the tracking task and the n-back task both need the visual module to perceive the stimuli. Given that the visual module can only process one request at a time, these tasks will have to wait for each other, resulting in a decrease in performance on both tasks. Combining tracking and tone counting, on the other hand, does not lead to significant interference for the model, because performance on those tasks is dependent on different modules (aural and problem state for counting; visual and manual for tracking). Finally, combining tone counting and the n-back task will result in the most severe predicted interference. Both tasks require the problem state module to keep track of the count and the letters, respectively. It has been shown that the problem state module can only maintain a single chunk of information, and that it causes considerable interference when required by two tasks concurrently [44,68,69]. The idea is that the contents of the problem state module—representing working memory of the first task—are stored in declarative memory when the other task needs to use the problem state. When resuming the first task this information has to be retrieved from declarative memory, which takes time and can go wrong, leading to increased RTs and error rates.

The model was not fit to the current data-set, we used the parameter settings from [64], both for the behavioral as well as for the neural predictions.

### Behavioral results

**Fig 7:**
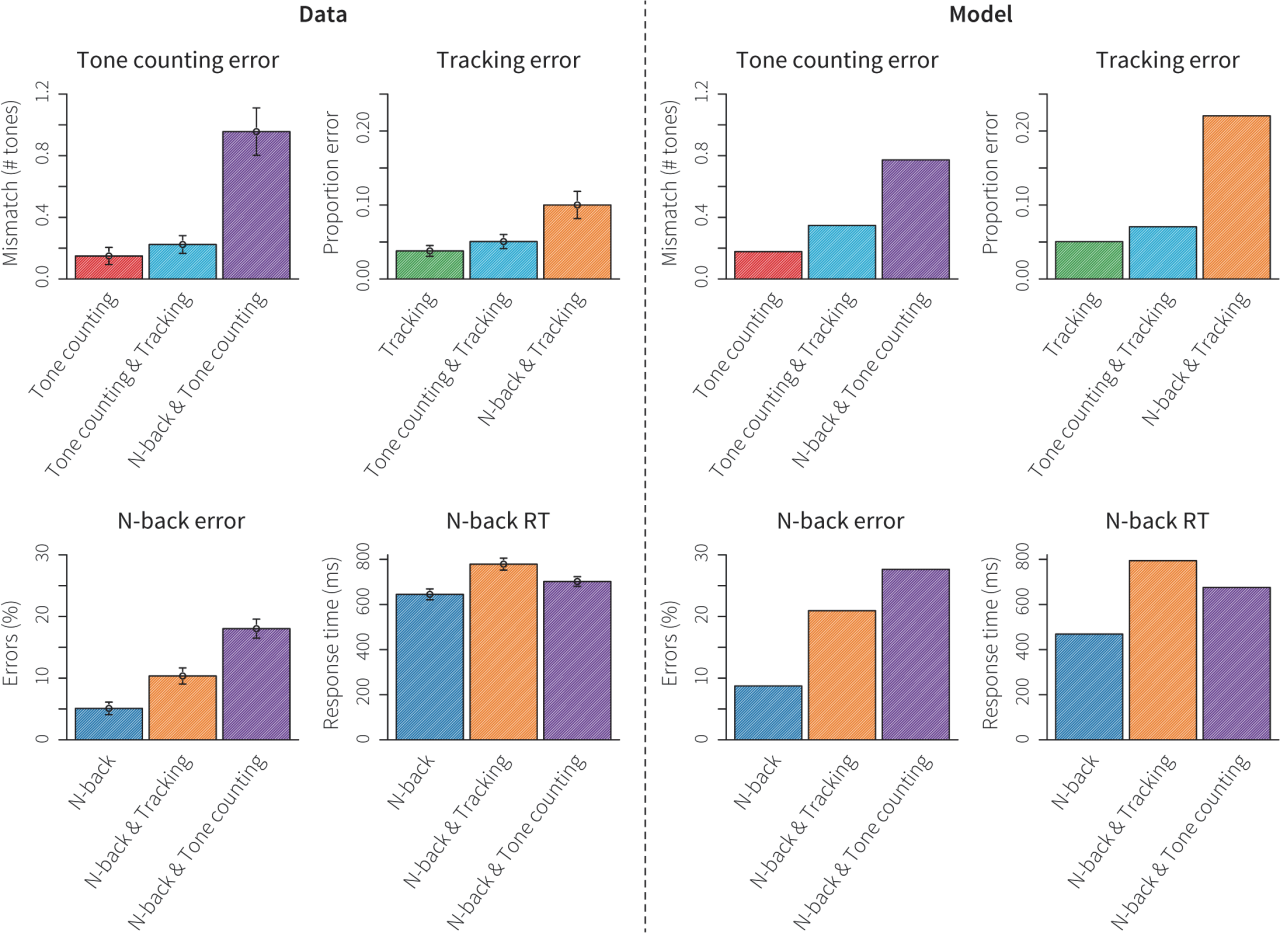
Behavioral results for the multitasking dataset. Left four graphs show the data, the right four graphs the model predictions. Error bars indicate standard error.

Fig. 7 shows the behavioral results: on the left the data, on the right the model predictions. The top left graph shows the mismatch between high tones presented and counted in the tone-counting task. Subjects performed well in all conditions, but made more errors when tone counting was combined with n-back, while tracking only had a minimal impact. The second graph shows the proportion of error time in the tracking task, which is the proportion of time the cursor was outside the vertical lines flanking the target dot. Subjects also performed very well on this task; only the combination with n-back led to a clear decrease in performance. The model predicted these results fairly accurately, although it predicted a much higher tracking error than displayed by the human subjects. In general, doing either of the tasks in combination with n-back led to the largest performance decrements. The model attributed those decrements to competition for the problem state module in the case of tone counting and to competition for the visual module in the case of the tracking task.

The bottom graphs of Fig. 7 show the results on the n-back task itself, percent error on the left and response time on the right. Interestingly, error rate increased most in combination with tone counting, while the combination with tracking led to the highest RTs. The model matched those effects qualitatively. It explained the increased error rate in combination with tone counting by competition for the problem state module. Because the contents of the problem state have to be swapped out via declarative memory for those tasks, incorrect letters are sometimes retrieved from memory, leading to errors on the n-back task. The increase in RT when n-back was combined with the tracking task was explained by competition for the visual resource: the model had to check the status of the tracking task regularly, resulting in delayed reactions to the n-back task.

As the purpose of the second dataset is to test the applicability of data-driven model-brain mappings when cognitive models are used to predict data *a priori*, we refrained from optimizing the model to fit the behavioral data as that would refute the notion of a true *a priori* prediction.

### Imaging results

**Fig 8:**
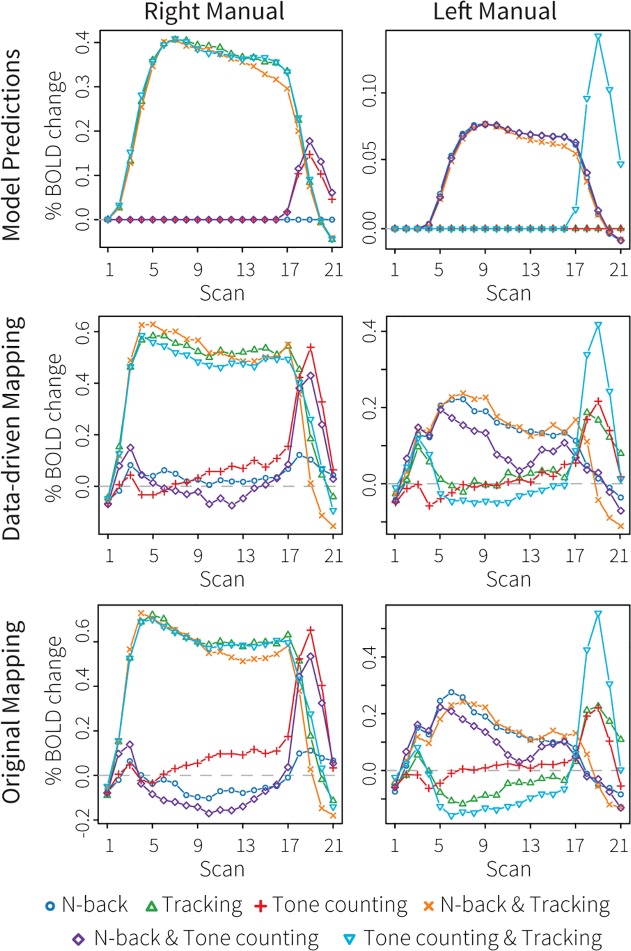
Imaging results of the multitasking dataset for the left and right manual modules. The top row shows model predictions, the middle row the BOLD responses in the new, data-driven ROIs, and the bottom row the BOLD responses in the original ROIs.

**Fig 9:**
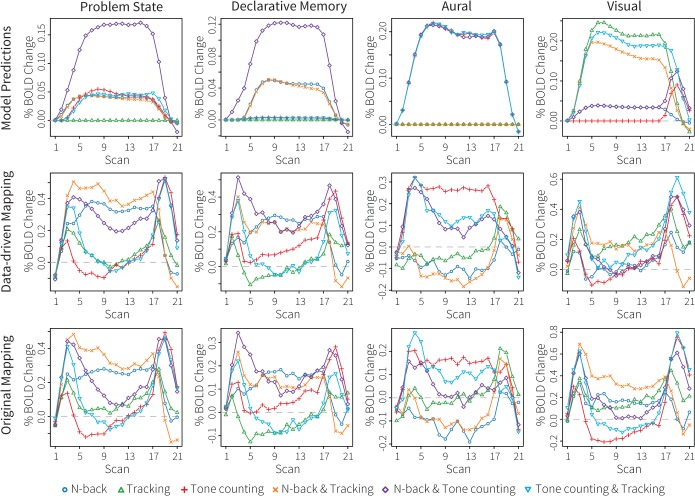
Imaging results of the multitasking dataset for the problem state, declarative memory, aural, and visual modules. The top row shows model predictions, the middle row the BOLD responses in the new, data-driven ROIs, and the bottom row the BOLD responses in the original ROIs.

Fig. 8 (left and right manual module) and Fig. 9 (problem state, declarative memory, aural, and visual modules) show the fMRI results. The model predictions are shown in the top rows of the figures, the results of the new data-driven ROIs in the middle rows, and the results of the original ROIs in the bottom rows. The colors of the conditions correspond to the colors in Fig. 7. Table 3 reports fit measures.

**Table 3:** Fit measures for the data-driven and original mappings on the multitasking dataset.

	Data-driven mapping				Original Mapping			
Module	TCC	Ind. TCC	R^2^	RMSD	TCC	Ind. TCC	R^2^	RMSD
Problem State	.60	.48 (.21; .00 –.72)	.08	.23	.50	.36 (.25; −.08–.67)	.01	.20
Declarative Memory	.68	.48 (.24; −.19–.80)	.26	.18	.63	.31 (.40; −.56–.78)	.21	.10
Right Manual	.96	.87 (.06; .74 –.95)	.88	.14	.96	.87 (.06; −.74–.96)	.86	.20
Left Manual	.80	.49 (.23; −.02–.83)	.49	.09	.73	.46 (.23; .01–.84)	.47	.11
Aural	.78	.52 (.23; .07–.90)	.63	.09	.62	.36 (.27; −.24–.72)	.43	.10
Visual	.64	.42 (.33; −.29–.82)	.08	.16	.54	.38 (.20; .05 –.65)	.03	.25

*Note*: TCC = Tucker’s Congruence Coefficient, Ind. TCC = mean individual TCC (standard deviation, range), RMSD = root-mean-square deviation.

For the right manual module (associated with the left motor cortex; Fig. 8), the model predicted high activation levels throughout the trial for all conditions involving tracking. In addition, it predicted a peak at the end of the trial for the tone-counting task (single task and in combination with n-back). These predictions follow from the fact that subjects had to use their right hands for these tasks. Both the data-driven and the original ROIs confirmed this pattern of activity. The data-driven model-brain mapping resulted in a minimally better fit, probably because the conditions that did not use the right hand remained closer to baseline. For the left manual module the model predicted much less activity: only the n-back task required left-handed responses during the trial, and only the tone-counting task in combination with tracking at the end of the trial. Both ROIs confirmed these effects; the data-driven ROI again showed a slightly better fit than the original ROI. The biggest discrepancy between model and data was the lower activity level in the n-back and tone-counting condition than in the other n-back conditions. This decrease might be caused by subjects failing to give all responses in the n-back task when it was combined with tone-counting (1.7% misses vs. 0.9% misses in the other conditions), while the model never missed a response (although it made more mistakes in this condition, see Fig. 7).

Fig. 9 shows the results for the other modules. The model predicted similar patterns for the problem state and declarative memory modules: activity in all conditions involving n-back and tone-counting, with a major increase in the n-back and tone-counting condition. As explained above, this increase is due to a constant swapping out of the problem state module via declarative memory in the n-back and tone-counting condition. Both ROIs disconfirmed this prediction. Instead, they indicated a decreased use of the problem state module in the n-back and tone-counting condition (perhaps caused by missed n-back stimuli, analogous to the left manual results discussed above). In addition, the tone-counting task seemed not to use the problem state module at all (subjects might have employed subvocal rehearsal strategies instead, see [63]). Given that neither mapping matched the model predictions, the fit measures can tell us little about which one is preferred. However, visual inspection showed a better separation of the n-back and tone-counting conditions from inactive conditions in the data-driven ROI for the problem state. In addition, the inactive conditions remain closer to zero in the data-driven ROIs.

The model predicted equal involvement of the aural module (third column in Fig. 9) in all tone-counting conditions. The data showed that this was not the case, with the dual-tasks leading to less activity in the aural ROIs—probably linked to less attention for tone counting in these conditions. Again, the data-driven ROI showed to a clearer separation of the conditions, and maintained activity throughout the trial for the n-back and tone-counting condition. Although we do not have access to ground truth, it seems reasonable to assume that subjects kept listening to the tones in this condition given their performance (Fig. 7), which is thus better reflected by the data-driven ROI. Finally, the model predicted considerable activity for all tracking conditions in the visual region. For the n-back task much less activity was predicted, and no activity at all for the tone-counting single task. These predictions were confirmed in part by either ROI. The data-driven ROI showed more activity for the tracking condition than for all other conditions, but no activity in response to n-back. The ROI of the original mapping, on the other hand, showed activity for all condition involving n-back, but hardly at all for the tracking conditions (only the tracking single task resulted in some activation).

### Discussion

For the second dataset we made fMRI predictions based on a pilot dataset [64]. The model’s behavioral fit to the current dataset was good, especially taken into account that these were *a priori* predictions. However, although the behavioral fit might have led us to believe that the model is on the right track, the fMRI data revealed otherwise. While the model correctly predicted the patterns in the left and right manual regions and showed a rough fit to the data in the aural and visual regions, it predicted the main effects incorrectly in the problem state and declarative memory regions—the core cognitive components of the model. The data seem to indicate that subjects did not use their working memory to perform the tone-counting task, as the model assumed. Furthermore, even in regions where the model fit well to the fMRI data, there were hints in the data on how the model should be improved (e.g., decreased attention to auditory stimuli in the tone-counting dual-tasks). In addition to the module-specific mismatches, there seems to be a general dip in the data in many regions between the start and the end of the trial, which was not captured by the model. One possible explanation is that there was a saturation in the BOLD response that was not captured by our HRF (e.g., [27]).

Our main interest in the current paper is whether model-based fMRI is a suitable method for creating model-brain mappings. As in the first dataset, the data-driven ROIs performed as well or better than the original ROIs. Conditions for which no activation was predicted remained closer to baseline (the data-driven regions especially showed less deactivation), and conditions were often better separated in the data-driven ROIs (problem state, aural). As in the first dataset, the visual ROIs showed the greatest differences. This dataset seemed to hint at a separate functionality of the two visual ROIs. The new, data-driven ROI reacted exclusively to the visual tracking task, whereas the original ROI responded more strongly to the n-back task. These results seem to indicate that the data-driven ROI is involved in visual-spatial processing, while the original ROI is more involved in detailed processing of the letters in the n-back task. This is in agreement with the literature on regional functions: the data-driven ROI is located in the occipital gyrus, part of the dorsal ‘where’ stream of visual processing, whereas the original ROI is located in the fusiform gyrus, part of the ventral ‘what’ stream (e.g., [70,71]).

## General Discussion

More and more researchers turn to neuroscience for constraints on formal models of cognition (e.g., [9–12]). Our second dataset illustrates why: even though we were able to predict the main patterns in the behavioral data—an important model requirement [2,5–7]—the neuroimaging data showed that several assumptions underlying the model were incorrect. However, before one can use neuroimaging data to constrain cognitive models, a mapping from model components to brain regions is needed. Originally, such a mapping was based on the experience of the researcher or on a reading of the literature, and was thus necessarily subjective. In this paper we proposed a more objective method to create such a mapping: model-based fMRI analysis (e.g., [22,23]). Model-based fMRI is a formal and data-driven method, and is therefore preferred over the original approach—at least if the results are comparable. To test whether model-based fMRI is indeed suitable we used it to create a mapping for five modules of the ACT-R cognitive architecture. We subsequently applied this new mapping, as well as the original, literature-based mapping, to two datasets: a relatively simple algebra task and a more demanding multitasking experiment. For each dataset we developed a model, and used this model to generate fMRI predictions. These predictions were compared to the data of both the original and the new, data-driven mapping.

We started with the algebra dataset because we have extensive experience with modeling algebra tasks in general and pyramid experiments in particular [55–58]. We could therefore be reasonably sure that our model of this task would match the data, thereby providing validation of the approach. Indeed, the fit to both the behavioral data and the fMRI data was good. The new data-driven model-brain mapping performed as well as the original mapping on this dataset, indicating that model-based fMRI is a suitable way to create model-brain mappings. As it is a more objective method than the original approach, it is therefore to be preferred, even though the results were not clearly better than the results of the original mapping.

The drawback of using such a simple task was that demands on the model components were limited and that differences between the two mappings might not have become apparent. We therefore used a much more challenging multitasking dataset as our second test. Instead of fitting data we made *a priori* fMRI predictions for this dataset. We did not expect to match all data in this case; instead, our aim was to provide an example of how fMRI data can be used to inform cognitive models (using the model as a ‘sacrificial lamb’ [9]). Indeed, even though the model matched the patterns in the behavioral data and in the perceptual and motor regions of the brain, its predictions for the cognitive components were incorrect. The results indicated which assumptions of the model need to be altered, and additionally showed that behavior alone does not provide sufficient constraints for models of complex cognition. Furthermore, the second dataset resulted in slightly more pronounced differences between the two mappings. In general, the data-driven model-brain mapping yielded a better separation between conditions of the experiment, and showed less activation and less deactivation for conditions that presumably did not involve the associated model components. As a result, it will provide better constraints and guidance for new models developed in the ACT-R architecture.

Although we demonstrated model-based fMRI in combination with the ACT-R architecture, it can be used in combination with a variety of cognitive models. The method was originally used in combination with mathematical models, which yield parameter values that typically vary on a trial-by-trial basis. These parameter values can be convolved with an HRF, and subsequently be used as regressors in the analysis. Such models have been used to locate the neural correlates of, for instance, reinforcement learning, category learning, and decision making [29–35]. In 2011, we have shown that model-based fMRI can also be used in combination with process models that make time-by-time predictions, such as ACT-R [36]. In fact, the method will work in combination with any formal model, as long as the model predicts activity of model components, either differentiating in temporal profile or in predicted amplitude between conditions (and preferably between trials or at least trial types, [36]). However, we are not aware of another modeling formalism that has used the results of model-based fMRI to create a mapping between model components and brain regions in order to constrain future models.

When using model-based fMRI to develop model-brain mappings, one has to be careful not to apply the method in a circular fashion. That is, one has to use one model (or, preferably, several models) to identify the model-brain mapping, and then use this mapping to confirm or disconfirm *different* models, as we did in the current paper. The same model can never be used to create the mapping and to be tested with the created mapping.

Given the small differences between the results of the data-driven model-brain mapping and the original mapping, one might wonder whether we need model-based fMRI to create such a mapping. The original mapping was based on the literature on regional functions and on the experience of the ACT-R research group. Even though it was very successful and turned out to be very close to our new data-driven model-brain mapping, it was therefore necessarily subjective. For that reason we argue that model-based fMRI is the preferred method, as it is data-driven and objective (although the models are still dependent on the researchers, a problem we circumvented by using the results of a meta-analysis). The current results can thus be seen as a confirmation of the original mapping. In addition, the new data-driven mapping was slightly more powerful than the original mapping. One reason for this might be that the new mapping follows brain structures, unlike the original mapping (see Fig. 4). Assuming that brain functions in general do not cross structural boundaries, this should lead to less noise in the signal.

Taking the results of the two datasets in to account, it seems clear that the new mapping of the problem state, declarative memory, aural, and manual modules is at least as powerful as the original mapping. However, the results of the visual module are less straightforward. In the first dataset the new mapping performed slightly better than the original mapping, under the assumption that the generating model was correct. In the second dataset, however, the new visual ROI exclusively reacted to the tracking task and not to the n-back task—indicating that it is involved in visual-spatial processing and not in processing of detailed stimuli. The original ROI, on the other hand, hardly responded to the tracking task, but more to the n-back task. These results are in agreement with the literature, which suggests that the location of the new ROI (the occipital gyrus) is involved in spatial processing, while the location of the original ROI is involved in reading and processing of detailed stimuli [43,70,71]. The functionality of the original ROI seems therefore closer to the functionality of the visual module in ACT-R, which is involved in encoding the details of the visual world. ACT-R currently does not have a module that is used for spatial reasoning. Although the visual-location module might be considered a spatial module, it only processes the location of objects on the screen, and does not perform any kind of spatial reasoning. In the current two datasets it matched worse to both the new and to the original visual ROI than the visual module. The current results imply that a visual-spatial module should be implemented and that its activity should be mapped onto the occipital gyrus.

## Supporting Information

S1 Fig Comparison of Tucker’s Congruence Coefficient (TCC) to R. (EPS)
